# RpuS/R Is a Novel Two-Component Signal Transduction System That Regulates the Expression of the Pyruvate Symporter MctP in *Sinorhizobium fredii* NGR234

**DOI:** 10.3389/fmicb.2022.871077

**Published:** 2022-04-29

**Authors:** Ana Laura Ramos, Maria Aquino, Gema García, Miriam Gaspar, Cristina de la Cruz, Anaid Saavedra-Flores, Susana Brom, Ramón Cervantes-Rivera, Clara Elizabeth Galindo-Sánchez, Rufina Hernandez, Andrea Puhar, Andrei N. Lupas, Edgardo Sepulveda

**Affiliations:** ^1^CELM, Ensenada, Mexico; ^2^Facultad de Ciencias, Universidad Autónoma de Baja California, Ensenada, Mexico; ^3^Facultad de Biología, Universidad Autónoma de Sinaloa, Culiacan, Mexico; ^4^Programa de Ingeniería Genómica, Centro de Ciencias Genómicas, Universidad Nacional Autónoma de México, Cuernavaca, Mexico; ^5^Departamento de Biotecnología Marina, Centro de Investigación Científica y de Educación Superior de Ensenada (CICESE), Ensenada, Mexico; ^6^The Laboratory for Molecular Infection Medicine Sweden (MIMS), Umeå University, Umeå, Sweden; ^7^Umeå Centre for Microbial Research (UCMR), Umeå University, Umeå, Sweden; ^8^Department of Molecular Biology, Umeå University, Umeå, Sweden; ^9^Departamento de Microbiología, Centro de Investigación Científica y de Educación Superior de Ensenada (CICESE), Ensenada, Mexico; ^10^Department of Protein Evolution, Max Planck Institute for Biology, Tübingen, Germany; ^11^CONACYT-Departamento de Microbiología, Centro de Investigación Científica y de Educación Superior de Ensenada (CICESE), Ensenada, Mexico

**Keywords:** TCSTSs, two-component signal transduction systems, piruvate, STAC, *Sinorhizobium fredii*

## Abstract

The SLC5/STAC histidine kinases comprise a recently identified family of sensor proteins in two-component signal transduction systems (TCSTS), in which the signaling domain is fused to an SLC5 solute symporter domain through a STAC domain. Only two members of this family have been characterized experimentally, the CrbS/R system that regulates acetate utilization in *Vibrio* and *Pseudomonas*, and the CbrA/B system that regulates the utilization of histidine in *Pseudomonas* and glucose in *Azotobacter*. In an attempt to expand the characterized members of this family beyond the Gammaproteobacteria, we identified two putative TCSTS in the Alphaproteobacterium *Sinorhizobium fredii* NGR234 whose sensor histidine kinases belong to the SLC5/STAC family. Using reverse genetics, we were able to identify the first TCSTS as a CrbS/R homolog that is also needed for growth on acetate, while the second TCSTS, RpuS/R, is a novel system required for optimal growth on pyruvate. Using RNAseq and transcriptional fusions, we determined that in *S. fredii* the RpuS/R system upregulates the expression of an operon coding for the pyruvate symporter MctP when pyruvate is the sole carbon source. In addition, we identified a conserved DNA sequence motif in the putative promoter region of the *mctP* operon that is essential for the RpuR-mediated transcriptional activation of genes under pyruvate-utilizing conditions. Finally, we show that *S. fredii* mutants lacking these TCSTS are affected in nodulation, producing fewer nodules than the parent strain and at a slower rate.

## Introduction

Two-component signal transduction systems (TCSTS) are regulatory systems that allow bacteria to respond to environmental stimuli by regulating different cellular processes like chemotaxis, nutrient acquisition and utilization, virulence, and antibiotic resistance. As their name implies, they are comprised of two elements, a histidine kinase that acts as the sensing and signal transducing component, and a response regulator, that usually activates or represses gene expression (Ann M. Stock et al., 2000; Tiwari et al., 2017). Most histidine kinases are membrane-bound proteins, detecting signals through extracellular sensory domains, which trigger a conformational change that is propagated across the membrane to the intracellular region of the protein, very often *via* a HAMP domain (Hulko et al., 2006). The conformational change induces the phosphorylation of a conserved histidine in the dimerization and histidine phosphotransfer domain (DHp) by the catalytic ATP-binding domain (CA). In many kinases, this process has additional levels of regulation by the action of other cytoplasmic domains, such as PAS and GAF (Gao and Stock, 2009). Finally, the phosphate group is transferred to a conserved aspartate in the receiver domain (REC) of response regulator proteins, translating the phosphorylation signal into a physiological response, often by modulating transcription (Galperin, 2010). In hybrid histidine kinases, a REC domain occurs on the same polypeptide with the core histidine kinase. This arrangement, that often is accompanied by the addition of extra elements in the phosphorelay, is believed to provide extra-fine-tuning mechanisms to the regulatory pathways involved (Borland et al., 2016).

CbrA and CrbS are histidine kinases belonging to a family of membrane-bound TCSTS sensor proteins in which the cytosolic histidine kinase domains are fused to a transmembrane SLC5 solute symporter domain through a STAC domain (Henriquez et al., 2021). The STAC domain is a recently described protein domain associated with bacterial signal transduction. Although its role is still unknown, it has been speculated that it could mediate interactions with other proteins or regulate the flow of substrates through the SLC5 domain (Korycinski et al., 2015).

CbrA is a histidine kinase that has been characterized as a global regulator of metabolism in the *Pseudomonadaceae*. Together with its cognate response regulator, CbrB, it regulates the utilization of histidine in *P. fluorescens* and *P. putida* (Zhang and Rainey, 2008; Zhang et al., 2015; Monteagudo-Cascales et al., 2019), and glucose in *Azotobacter vinelandii* (Quiroz-Rocha et al., 2017). Interestingly, it has been found that neither is the kinase domain of CbrA required for histidine uptake by the SLC5 domain nor the SLC5 domain essential for signal perception, strongly suggesting that stimulus sensing in this family occurs in the cytoplasm (Monteagudo-Cascales et al., 2019; Wirtz et al., 2020). Recently, it has been shown in *P. putida* that *cbrX*, a small ORF that is co-transcribed with *cbrA*, modulates CbrA translation by a mechanism of translational coupling (Monteagudo-Cascales et al., 2019).

CrbS is a hybrid histidine kinase, which regulates acetate utilization in *Vibrio cholerae* and the *Pseudomonadaceae* (Jacob et al., 2017; Henriquez and Jung, 2021). CrbR, its cognate response regulator, is a LuxR transcriptional activator that, in Gammaproteobacteria, induces expression of acetate utilization genes by binding to a conserved DNA sequence motif in their promoter region (Sepulveda and Lupas, 2017). Recently, it has been demonstrated that in *V. cholerae* the cAMP receptor protein (CRP)-cAMP system activates the expression of the *crbS* and *crbR* genes (Muzhingi et al., 2018).

Although SLC5/STAC-containing signaling proteins can be found among all phyla of bacteria (Korycinski et al., 2015), the work on this TCSTS family has been restricted mainly to the Gammaproteobacteria. Only the PrlS/PrlR system from *Brucella melitensis*, which plays a role in bacterial adaptation to ionic strength and persistence in mice (Mirabella et al., 2012), has been genetically characterized outside this phylum. In this work, we identified the CrbS/R system in the Alphaproteobacterium *Sinorhizobium fredii* and determined that, as in the Gammaproteobacteria, it is involved in acetate utilization. Moreover, we report the characterization of RpuS from *Sinorhizobium fredii*, which together with RpuR forms a two-component system that is required for optimal growth on pyruvate as the sole carbon source. Using RNAseq, we identified a plasmid-encoded sodium-solute symporter as its primary target and identified the minimal promoter region required for RpuS/RpuR regulation. Finally, we determined the effect that a deletion of this system has on nodulation.

## Materials and Methods

### Growth Conditions

*Sinorhizobium fredii* and *Escherichia coli* strains were grown in Peptone-Yeast medium (PY; Bravo and Mora, 1988). *S. fredii* NGR234 was obtained from the Culture Collection of the Centro de Ciencias Genómicas-UNAM. Experiments that required a defined carbon source were performed in Minimal Media (MM; NaNO_3_ 2.5 g/l; KH_2_PO_4_ 1 g/l; K_2_HPO_4_ 2.0 g/l; MgSO_4_-7H_2_O 0.3 g/l; NaCl 0.1 g/l; FeCl_3_ 0.01 g/l) supplemented with 1 ml/l of Nitsch’s trace elements (Atlas, 2010), and a carbon source at a final concentration of 20 mm. When required, antibiotics were added at the following concentrations: gentamicin (Gm), 25 μg/ml; kanamycin (Km), 30 μg/ml; nalidixic acid (Nal), 20 μg/ml; rifampicin (Rif), 25 μg/ml. For selection against pK18mobSacB, PY agar plates were supplemented with 10% sucrose. Genes cloned in pSRKgm were induced by the addition of 100 μm of isopropyl-β-D-thiogalactopyranoside (IPTG).

Pre-cultures for assays in MM were prepared in 5 ml PY and washed with MM as described previously (Zhang et al., 2015). Growth curves were performed by incubating 25 ml cultures at 30°C in an orbital shaker incubator at 150 rpm and monitored by reading 100 μl samples using a Multiskan Sky plate reader and the SkanIt 6 software (Thermo Fisher, Waltham, United States). Replicates for growth curves, transcriptional fusion measurements, and RNAseq are biological, derived from independent cultures.

### Plasmid and Strain Construction

Bacterial strains and plasmids used in this study are listed in Table 1; primer sequences are listed in Supplementary Table S1. All molecular techniques were conducted following standard protocols (Sambrook et al., 2012). *S. fredii* NGR234 (Stanley et al., 1988) was transformed by electroporation as described previously (Ferri et al., 2010). All constructs were verified by sequencing (Eton Bioscience, Inc., Sand Diego, United States) and all mutants were confirmed by PCR.

**Table 1:** Strains and plasmids used in this work.

Strain	Relevant phenotype	References
*Sinorhizobium fredii*		
NGR234	Parent strain Nal^r^ Rif^r^	Stanley et al., 1988
NGR Δ12300	*ΔcrbS* Nal^r^ Rif^r^	This work
NGR Δ12295	*ΔcrbR* Nal^r^ Rif^r^	This work
NGR Δ10960	*ΔrpuS* Nal^r^ Rif^r^	This work
NGR Δ10955	*ΔrpuR* Nal^r^ Rif^r^	This work
NGR ΔDbl	*ΔcrbS ΔrpuS* Nal^r^ Rif^r^	This work
NGR ΔcrbRc	NGR Δ12295 derivative with pSRKgm-crbR Nal^r^ Rif^r^ Gm^r^	This work
NGR ΔrpuRc	NGR Δ10955 derivative with pSRKgm-rpuR Nal^r^ Rif^r^ Gm^r^	This work
NGR ΔmctP	*ΔmctP* Nal^r^ Rif^r^	This work
NGR Δ10970	*ΔNGR_RS10970* Nal^r^ Rif^r^	This work
NGR PrMctP	NGR234 derivative with integrated *pVMG-PrmctP* Nal^r^ Rif^r^ Km^r^	This work
NGR ΔrpuR prMctP	NGR Δ10955 derivative with integrated *pVMG-PrmctP* Nal^r^ Rif^r^ Km^r^	This work
NGR ΔrpuRc prMctP	NGR Δ10955 derivative with pSRKgm-rpuR and *pVMG-PrmctP* Nal^r^ Rif^r^ Gm^r^ Km^r^	This work
NGR PrMctPM2	NGR234 with integrated *pVMG-PrmctPM2* Nal^r^ Rif^r^ Km^r^	This work
NGR ΔrpuR PrMctPM2	NGR Δ10955 with integrated *pVMG-PrmctPM2* Nal^r^ Rif^r^ Km^r^	This work
NGR ΔrpuRc PrMctPM2	NGR Δ10955 derivative with pSRKgm-rpuR and *pVMG-PrmctPM2* Nal^r^ Rif^r^ Gm^r^ Km^r^	This work
**Plasmids**	**Relevant phenotype/use**	**References**
pSRKgm	Broad-host expression vector Gm^r^	Khan et al., 2008
pSRKg-crbR	Expression of *crbR* in *S. fredii* Gm^r^	This work
pSRKg-rpuR	Expression of *rpuR* in *S. fredii* Gm^r^	This work
pSRKg-crbS	Expression of *crbS* in *S. fredii* Gm^r^	This work
pSRKg-rpuS	Expression of *rpuS* in *S. fredii* Gm^r^	This work
pBBR53gus	*uidA* transcriptional fusion plasmid Gm^r^	Girard et al., 2000
pBBR53gus-Pr*mctP*	*uidA* transcriptional fusion of the *mctP* operon promoter derivative Gm^r^	This work
pBBR53gus-Pr*mctP* + 18	pBBR53gus-Pr*mctP* + 18 derivative Gm^r^	This work
pBBR53gus-Pr*mctP*-20	pBBR53gus-Pr*mctP* -20 derivative Gm^r^	This work
pBBR53gus-Pr*mctP-75*	pBBR53gus-Pr*mctP* -75 derivative Gm^r^	This work
pBBR53gus-Pr*mctP*-82	pBBR53gus-Pr*mctP* -82 derivative Gm^r^	This work
pBBR53gus-Pr*mctP*-30	pBBR53gus-Pr*mctP* -30 derivative Gm^r^	This work
pBBR53gus-Pr*mctP*-40	pBBR53gus-Pr*mctP* -40 derivative Gm^r^	This work
pVMG	*uidA* transcriptional fusion integrative plasmid Km^r^	Gao et al., 2019
pVMG-Pr*mctP*	*Cis uidA* integrative transcriptional fusion of the *mctP* operon promoter derivative Km^r^	This work
pVMG-Pr*mctP*mut2	pVMG-Pr*mctP* mut2 derivative Km^r^	This work
pK18mobSacB	Allelic exchange vector Km^r^ Sac^s^	Schäfer et al., 1994
pK18mobSacB-del10970	Deletion of *NGR_RS10970* Km^r^ Sac^s^	This work
pK18mobSacB-delcrbS	Deletion of *crbS* Km^r^ Sac^s^	This work
pK18mobSacB-delcrbR	Deletion of *crbR* Km^r^ Sac^s^	This work
pK18mobSacB-delrpuS	Deletion of *rpuS* Km^r^ Sac^s^	This work
pK18mobSacB-delrpuR	Deletion of *rpuR* Km^r^ Sac^s^	This work
pK18mobSacB-delmctP	Deletion of *mctP* Km^r^ Sac^s^	This work

Strains with deletions in specific genes were constructed by allelic exchange and selected by sucrose sensitivity, using plasmid pK18mobSacB (Schäfer et al., 1994). The pK18mobSacB derivatives for the deletion of *rpuS* and *crbS* were constructed by overlap extension (Lee et al., 2010) of two PCR products corresponding to ~750 bp of the upstream and downstream sequence of the targeted gene, and the resulting product was re/amplified and cloned in the HindIII/EcoRI and HindIII/SmaI sites of plasmid pK18mobSacB, respectively. The pK18mobSacB derivatives for the deletion of *rpuR*, *cbrR, NGR_RS10965, and mctP* were obtained by cloning in the HindIII/EcoRI sites of plasmid pK18mobSacB a PCR product containing the gene to be deleted, together with ~750 bp of upstream and downstream sequence. Then the targeted gene was deleted by total PCR of the plasmid with phosphorylated divergent primers (Imai et al., 1991). The resulting PCR product was ligated, and the religated plasmid was recovered by transformation.

Plasmids pSRKgm-*rpuR and* pSRKgm-*crbR* were built by cloning the ORF of each gene into the NdeI/BamHI sites of plasmid pSRKgm (Khan et al., 2008). Plasmids pSRKgm-*rpuS and* pSRKgm-*crbS* were built by cloning the ORF of each gene in plasmid pSRKgm by Gibson cloning using the GeneArt Gibson Assembly HiFi Master Mix (Thermo Fisher, Waltham, United States).

The *in-cis uidA* transcriptional fusion of the *mctP* operon was constructed by cloning a PCR product of the putative promoter region in the SalI/BamHI sites of plasmid pVMG (Gao et al., 2019) producing plasmid pVMG-Pr*mctP*. Mutagenesis of the *mctP* operon promoter was achieved by the quick-change strategy (Agilent, Santa Clara, United States) using plasmid pVMG-Pr*mctP* as a template. After electroporation, plasmid cointegration and orientation were verified by PCR as described previously (Gao et al., 2019).

The *in-trans uidA* transcriptional fusion of the *mctP* operon promoter was constructed by cloning a PCR product of the putative promoter region in the BcuI/BamHI sites of plasmid pBBR53Gus (Girard et al., 2000). Transcriptional fusions used to map the NGR_RS10970 operon promoter were built by amplifying plasmid pBBR53g-Pr*mctP* with divergent primers carrying a BcuI site or a BamHI site. The resulting PCR product was digested with the corresponding restriction enzyme, ligated, and transformed for the recovery of the reconstituted plasmid.

### RNA-Sequencing and Bioinformatic Analysis

Two 20-ml cultures of the appropriate strains were inoculated from independent pre-cultures to an A_620_ of 0.1 in minimal medium supplemented with pyruvate and incubated at 150 rpm and 30°C for 12 h. RNA was isolated from 500 μl aliquots using the RiboPure RNA Purification Kit for bacteria (Thermo Fisher Scientific, Waltham, United States) and RNA purity and integrity were checked by agarose gel electrophoresis. Then, to eliminate the residual DNA, the samples were treated with DNase-I using the RQ1 RNase-Free DNase Kit (Promega, Madison, United States).

The RNA samples treated with DNase-I were used to prepare high throughput sequencing (HTS) libraries. First, rRNA depletion was performed using the QIAseq FastSelect—5S/16S/23S Kit (Qiagen, Hilden, Germany). Then, the resulting depleted and fragmented mRNA was used as input for the first-strand cDNA synthesis step of the TruSeq Stranded Kit (Illumina, San Diego, United States) and sequenced on an Illumina MySeq platform at the CICESE Functional Genomics Laboratory facility (Ensenada, Baja California, México), with a paired-end protocol and read length of 75 nt (PE 2 × 75), resulting in a total output of approximately 3.6 to 5.2 million reads per sample.

All read outputs were checked to satisfy Illumina quality standards (Ewels et al., 2016; Wingett and Andrews, 2018). Raw data were cleaned up with trimmomatic/0.36 (Bolger et al., 2014) to remove sequences originating from Illumina adaptors and low-quality reads. Files were aligned to the reference genome [RefSeq accession numbers NC_012587 (chromosome), NC_000914 (plasmid a), and NC_012586 (plasmid b)] with bowtie2/2.3.5.1(Langmead and Salzberg, 2012) using -N 0 (no mismatches accepted in the alignment) and --no-unal (suppress SAM records for unaligned reads) settings. All the other settings were implemented with default options. Once the alignment was completed, samtools/1.12 (Li et al., 2009) was used to select the reads that were aligned in proper pairs, and to sort them. The reads counting per gene was performed with htseq/0.9.1 (Anders et al., 2014) using stranded mode and gene as a feature. Differential expression analysis was performed with DESeq2/1.28.1(Love et al., 2014). Genes with adjusted *p*-values <0.05 and log2 fold changes ≥1 were considered differentially expressed. Differential expression plots were prepared in R/4.0.3 (R Core Team, 2021) using the packages ggplot2/3.2.5 (Wickham and Sievert, 2016) and EnhancedVolcano/1.6.0 (Blighe and Lewis, 2021).

### β-Glucuronidase Assays

Five-milliliter cultures of the appropriate strains were inoculated from three independent pre-cultures to an A_620_ of 0.1 in MM supplemented with succinate or pyruvate and incubated at 30°C for 8 h. β-glucuronidase activity expressed in modified Miller units was quantified as described previously (Jefferson et al., 1986). Photometric measurements were performed with a Multiskan Sky plate reader (Thermo Fisher, Waltham, United States).

### Nodulation Assays

Common bean *Phaseolus vulgaris* cv. Negro Jamapa seeds were surface-sterilized by sequential immersions in 96% (v/v) ethanol for 30 s and 6% sodium hypochlorite for 7 min. Seeds were subsequently rinsed several times with sterilized water and placed for germination on plates containing 1% water agar at 30°C for 48 h in the dark. Nodulation kinetic assays were performed as described previously (Rodríguez et al., 2020). Briefly, 2-day-old seedlings were transferred to growth pouches (CYGTM) containing 10 ml of nitrogen-free Fahräeus nutrient solution. Bacterial inocula were grown overnight in PY liquid medium, concentrated by centrifugation, and washed once in a solution of MgSO_4_ 10 mm. Bacterial suspensions were adjusted at an A_600_ of 0.3 and seedlings were inoculated by direct addition to each root of appropriate amounts of the bacterial solution, to give a final bacterial density of 10^8^ cells ml − 1 in the growth pouch. Pouches were kept under controlled environmental conditions (14/10-h light/dark cycle, 22°C/16°C, and 60 to 70% relative humidity). For each bacterial strain, eight growth pouches, containing one seedling each, were monitored daily for nodule appearance and the number of nodules was registered daily for 30 days.

### Bioinformatics and Statistical Analysis

The global search for SLC5/STAC proteins was performed as described previously (Korycinski et al., 2015). GeConT (Martinez-Guerrero et al., 2008) was used to search for homologs of the *rpuR* and *rpuS* to retrieve their putative promoter region. MEME (Bailey et al., 2009) and FIMO (Grant et al., 2011) searches were run with the default options searching for a motif between 6 and 30 nucleotides long. Sequence alignments and analyses were performed in the MPI bioinformatics toolkit (Zimmermann et al., 2018; Gabler et al., 2020) using MUSCLE (Edgar, 2004), Quick2D, HHpred (Hildebrand et al., 2009), PCOILS (Lupas et al., 1991), and MARCOIL (Delorenzi and Speed, 2002). Transmembrane segments were predicted with FMHMM (Sonnhammer et al., 1998) and Phobius (Käll et al., 2004). For transcriptional fusions, statistical comparisons between samples were performed by ANOVA and Tukey’s multiple comparison test using SPSS version 25 (IBM).

### Availability of Data

The raw RNA-sequencing data are deposited in SRA under the accession numbers SRR16684185 and SRR16684186 for wild type, and SRR16684183 and SRR16684184 for mutant samples. The total reads and the differential expression analysis tables are available as Supplementary Tables S3 and S4, respectively.

## Results

### Identification of Two of SLC5/STAC Two-Component Systems in *Sinorhizobium fredii* NGR234

**Figure 1:**
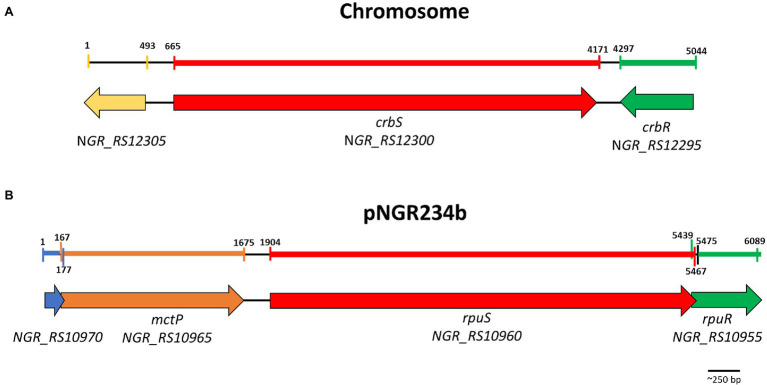
Genomic context of the CrbS/R and RpuS/R TCSTS. Genes are annotated as follows Red, SLC5/STAC Histidine kinase; Green, LuxR family response regulator; Orange, monocarboxylate permease; Blue, DUF3311 domain-containing protein; Yellow, Large-conductance mechanosensitive channels mscL family. Colored areas in the scale bars indicate the region deleted for the construction of the mutant strain in the corresponding gene.

A global search for SLC5/STAC proteins allowed us to identify two proteins belonging to this family in *Sinorhizobium fredii* NGR234, NGR_RS12300, and NGR_RS10960. Both are hybrid histidine kinases, with a REC domain at the C-terminal end as found in CrbR. The SLC5 domain is composed of 13 transmembrane segments, linked to a histidine kinase *via* STAC, then PAS, then a coiled-coil segment (Supplementary Figure S1). The *NGR_RS12300*/*NGR_RS12295* system is located on the chromosome. Both genes are adjacent but are transcribed convergent from each other and *NGR_RS12295* codes for a response regulator of the LuxR family, like CrbR. On the other hand, the *NGR_RS10960/NGR_RS10955* system is located on the pNGR234b megaplasmid. Both genes are predicted to form an operon, and gene *NGR_RS10955* also codes for a response regulator of the LuxR family (Figure 1).

### The CrbS/CrbR Two-Component System Is Required for Acetate Utilization in *Sinorhizobium fredii* NGR234

**Figure 2:**
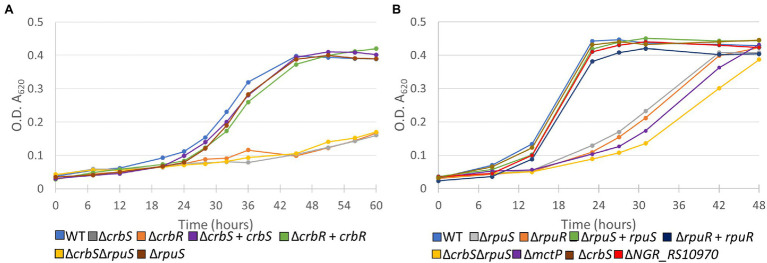
Growth curves of deletion mutants of **(A)** CrbS/R and **(B)** RpuS/R TCSTS. Bacteria were grown in minimal medium complemented with **(A)** acetate or **(B)** pyruvate as sole carbon sources. Medium was not complemented with Kanamycin in any case. Expression from plasmid pSRKgm was induced with 100 mm IPTG. Results are means for six independent cultures. Empty pSRKgm controls for deleted mutants and non-induced controls for complemented strains were also tested but showed a negligible effect on growth phenotype and are not depicted.

To determine the biological role that NGR_RS12300 and its cognate response regulator play in *S. fredii*, we first constructed a Δ*NGR_RS12300* mutant and screened it for its ability to grow in solid minimal media supplemented with different carbon sources. Our results showed that the Δ*NGR_RS12300* strain was unable to grow on acetate as the sole carbon source (Figure 2; Supplementary Figure S3). These results suggested that the *NGR_RS12300* and *NGR_RS12295* genes are the *S. fredii* homologs of CrbS and CrbR, respectively. To further test this hypothesis, we constructed Δ*NGR_RS12295* (ΔCrbR) strains that, as expected, was unable to grow on acetate. When both mutants were complemented, growth on acetate was successfully recovered (Figure 2A). Our results are congruent with the characteristics of the CrbS/R system in the Gammaproteobacteria, therefore we renamed *NGR_RS12300* and *NGR_RS12295* to *crbS* and *crbR*, respectively (Supplementary Table S2).

### Rpus/R Is Required for Optimal Growth in Pyruvate as the Sole Carbon Source in *Sinorhizobium fredii* NGR234

The putative *NGR_RS10960/NGR_RS10955* system was of particular interest to us because all the previously characterized two-component systems of the family are located on the chromosome, while this one is located on the pNGR234b megaplasmid. Located 220 bp upstream of NGR_RS10960 is another operon comprised of two genes, NGR_RS10965 and NGR_RS10970, of which NGR_RS10965 codes for a sodium:solute symporter family protein (Figure 1). This was evocative because in *P. fluorescens* the CrbR/S system regulates the *actP* gene, which also encodes a sodium-solute symporter family protein (Jacob et al., 2017) but this is not necessary for optimal growth in acetate (Sepulveda and Lupas, 2017). Rather, a BLASTP similarity search of NGR_RS10965 indicated that it is highly similar to the *Rhizobium leguminosarum* permease MctP, which transports alanine, pyruvate, and lactate, and is required for optimal growth on pyruvate as sole carbon source (Hosie et al., 2002). In *R. leguminosarum* and other species of the genus *Rhizobium, mctP* is regulated by the two-component system MctS/R. MctS is a transcriptional regulator integrated by a molecule-binding Cache2 domain and a Histidine kinase domain. These observations suggested to us that the NGR_RS10960 and NGR_RS10955 system is a new two/component system regulating *mctP*.

To test our hypothesis, we first constructed a Δ*NGR_RS10960* mutant and tested it for its ability to utilize alanine, pyruvate, and lactate as sole carbon sources. Our results showed that the mutant strain was able to grow on alanine and lactate but showed impaired growth on pyruvate (Supplementary Figure S2). Next, we constructed a Δ*NGR_RS10955* strain and compared its growth and that of *ΔNGR_RS10960* with that of the wild-type strain in liquid minimal medium supplemented with pyruvate. Our results showed that the parent strain reached the stationary phase ~24 h earlier than both mutants. Moreover, when both strains were complemented, growth rate was almost recovered to parent strain levels (Figure 2B). Overall, these results showed that the *NGR_RS10960* and *NGR_RS10955* constitute a two-component signal transduction system that is involved in the regulation of pyruvate uptake and therefore we renamed the genes to *rpuS* and *rpuR*, respectively (Supplementary Table S2). As expected, a double Δ*crbS*Δ*rpuS* mutant was not able to utilize acetate and showed growth retardation when growing on pyruvate (Figure 2; Supplementary Figure S2). Additionally, our results show that the Δ*crbS* strain is able to grow normally in pyruvate and the Δ*rpuS* mutant was able to utilize acetate. Thus, the two systems appear to operate independently without substantial crosstalk (Figure 2; Supplementary Figure S2).

**Figure 3:**
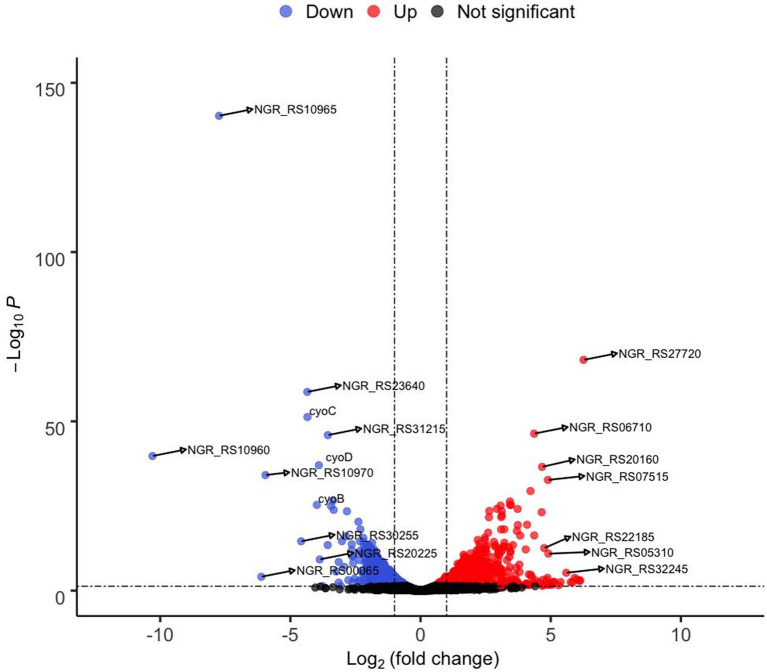
Volcano graph showing fold change in expression of genes in the Δ*rpuS S. fredii* strain compared to the parental strain (NGR234) when growing on pyruvate. Cultures were inoculated to a A_620_ of 0.1 in minimal medium supplemented with pyruvate and incubated at 150 rpm and 30°C for 12 h. RNA was isolated from 500 μl aliquots. Colored dots indicate upregulated (red) and downregulated (blue) genes. Labeled dots correspond to some of the genes listed in Table 2.

Interestingly, although the *rpuS* deletion also deleted the original ATG start-codon and ribosomal binding site (RBS) as well as the 5′-sequence of *rpuR* (Figure 2), *in-trans* complementation of the *rpuS* deleted strain was able to restore growth in pyruvate (Figure 3). Therefore, it can be concluded that in the *rpuS* strain *rpuR* is being translated from the SD of *rpuS* and that the missing amino acids are not essential for RpuR function.

### Defining the RpuS/R Regulon

To confirm if RpuS/R regulates the expression of *mctP* and to identify additional genes involved in pyruvate utilization that are regulated by this new system, we performed an RNAseq analysis to compare strains NGR234 (parent strain) and Δ*rpuS* after incubation for 12 h in minimal medium supplemented with pyruvate. Our results show a clear transcriptome profile specific to the mutant strain in a sample distance analysis against the parent strain (Supplementary Figure S3). The main characteristics are the repression of genes involved in energy generation and the induction of stress-related proteins and transmembrane transporters (Table 2; Figure 3). These results are consistent with the cells being under starvation, by the lack of a carbon source. Among other genes that show a small but not significant decrease in expression in the mutant strain when compared to the wild-type strain, we found *pycar* (Supplementary Table S4). This gene codes for the Pyruvate carboxylase enzyme (EC 6.4.1.1,) a central component of pyruvate metabolism that transforms pyruvate into oxaloacetate, which then can be fed into the TCA cycle to produce ATP (Dunn, 1998).

**Table 2:** Selection of differentially expressed genes in the *S. fredii* NGR234 Δ*rpuS* strain under pyruvate-utilizing conditions.

Supressed genes				
Gene ID	Fold change	Annotation	Function	Replicon
NGR_RS10965	−7.74	Sodium:solute symporter family protein	Transport	pNGR234b
NGR_RS00065	−6.11	Amino acid transporter	Transport	pNGR234b
NGR_RS10970	−5.95	DUF3311 domain-containing protein	Unknown	pNGR234b
NGR_RS30255	−4.58	Hemerythrin domain-containing protein	Energy	pNGR234a
NGR_RS23640	−4.35	Peroxiredoxin	Stress	Chromosome
cyoC	−4.34	Cytochrome bo(3) ubiquinol oxidase subunit 3	Energy	pNGR234b
cyoB	−3.98	Cytochrome bo(3) ubiquinol oxidase subunit 1	Energy	pNGR234b
cyoD	−3.90	Cytochrome bo(3) ubiquinol oxidase subunit 4	Energy	pNGR234b
NGR_RS20225	−3.87	Cbb3-type cytochrome c oxidase subunit 3	Energy	Chromosome
NGR_RS20200	−3.56	FixH family protein	Energy	Chromosome
NGR_RS31215	−3.56	OmpW family protein	Transport	pNGR234a
NGR_RS09030	−3.44	SURF1 family protein	Energy	pNGR234b
NGR_RS15265	−3.37	Hsp70 family protein	Stress	Chromosome
NGR_RS10955	−3.34	Response regulator transcription factor	Regulation	pNGR234b
queD	−3.25	6-carboxytetrahydropterin synthase	biosynthesis	pNGR234b
CyoA	−3.14	Cytochrome bo(3) ubiquinol oxidase subunit 2	Energy	pNGR234b
Induced Genes				
**Gene ID**	**Fold change**	**Annotation**	**Function**	**Replicon**
NGR_RS22030	3.74	ABC transporter substrate-binding protein	Transport	Chromosome
NGR_RS06025	3.79	NCS2 family permease	Transport	pNGR234b
NGR_RS21085	3.8	Hypothetical protein	Unknown	Chromosome
NGR_RS27445	3.83	RNA polymerase factor sigma-32	Stress	Chromosome
pobA	4.04	t 41 hydroxybenzoate hydroxylase	Transport	pNGR234b
NGR_RS05295	4.09	DUF2934 domain-containing protein	Unknown	pNGR234b
NGR_RS29265	4.22	Ferritin-like domain-containing protein	Stress	Chromosome
NGR_RS05300	4.36	CBS domain-containing protein	Regulation	pNGR234b
NGR_RS06710	4.36	CerR family C-terminal domain-containing protein	Regulation	pNGR234b
NGR_RS09255	4.65	DctP family TRAP transporter solute-binding subunit	Transport	pNGR234b
NGR_RS20160	4.67	Carbohydrate ABC transporter substrate-binding protein	Transport	Chromosome
NGR_RS22185	4.75	CsbD family protein	Stress	Chromosome
NGR_RS07515	4.89	Sugar ABC transporter substrate-binding protein	Transport	pNGR234b
NGR_RS05310	4.91	Hypothethycal membrane protein	Unknown	pNGR234b
NGR_RS32245	5.60	Hypothetical protein	Unknown	pNGR234b
NGR_RS27720	6.26	MFS transporter	Transport	Chromosome

Only those genes with differential expression values having values of *p*<10e^−5^ are listed.

Surprisingly, only the operon containing the genes *mctP* and *NGR_RS10970*, showed a relevant decrease in expression (Figure 3). To understand the role that these proteins have in pyruvate utilization we constructed deletion mutants of each of the genes and challenged them to grow on minimal medium supplemented with pyruvate. While the Δ*NGR_RS10970* mutation showed a negligible effect on growth, the Δ*mctP* strain displayed a ~ 24 h delay in reaching the stationary phase, just like the Δ*rpuS* and Δ*rpuR* strains (Figure 2B).

### An Imperfect Direct Repeat Is Required for RpuR-Mediated Transcriptional Activation

**Figure 4:**
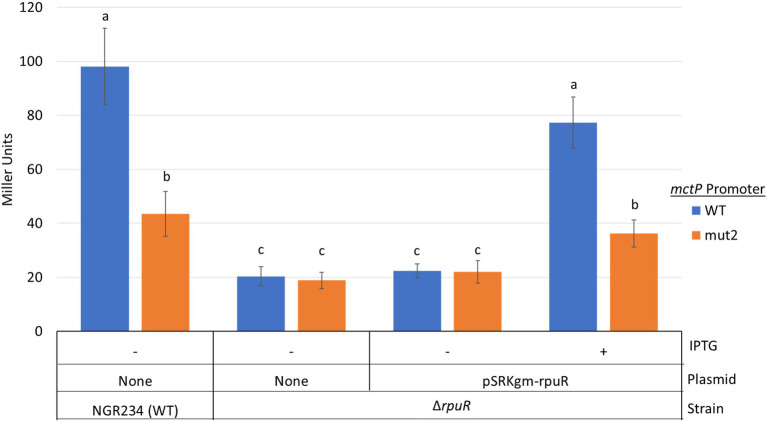
Activity of *in-cis* transcriptional fusions of the of the NGR_RS10970-*mctP* operon promoter, and its mut2 derivative, in various genetic backgrounds under pyruvate-utilizing conditions. Miller units are expressed as means ± Standard deviations (error bars) of results from at least three independent experiments. Means accompanied by the same letter are not significantly different (p < 0.05). NGR234, parent strain; ΔrpuR, *rpuR* deleted strain; +, induction of *rpuR* expression from plasmid pSRKgm with 100 mm IPTG. Cultures were inoculated to a A_620_ of 0.1 in minimal medium supplemented with pyruvate and incubated at 150 rpm and 30°C for 12 h.

**Figure 5:**
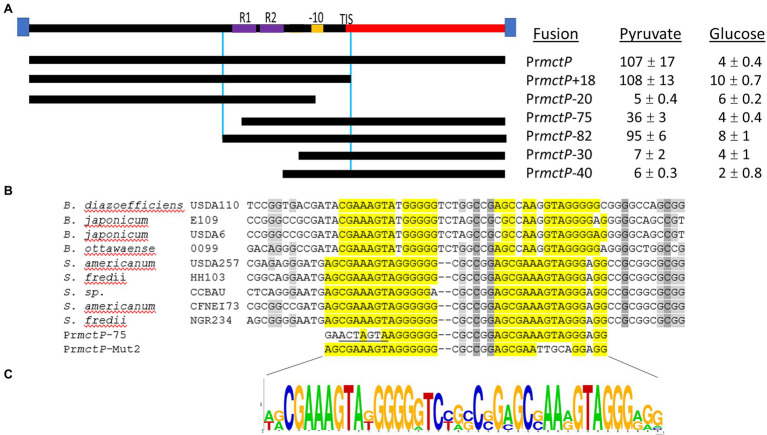
Mapping of the NGR_RS10970-*mctP* operon promoter. **(A)** Activities of *in-trans* transcriptional fusions of the NGR_RS10970-*mctP* operon promoter and its derivatives in a wild-type strain growing on pyruvate or glucose as sole carbon sources. Miller units are expressed as means ± Standard deviations of results from at least three independent experiments. **(B)** Sequence of the *mctP* operon promoter in different members of the Rhizobiaceae and of the *mctP* derivatives with mutations in the first (Pr*mctP*-75) and second (Pr*mctP*-Mut2) repeat. **(C)** Sequence Logo of the identified direct repeat obtained from the aligned sequences of the NGR_RS10970-*mctP* operon promoter of different members of the Rhizobiaceae.

Next, we studied the sequence properties of the RpuS/R-responsive *mctP* promoter. We used the RNAseq data to predict the transcription initiation site (TIS) to be located ~100 base pairs upstream from the first codon of *NGR_RS10970* (Cervantes-Rivera and Puhar, 2020). Approximately 22 bp upstream of the TIS lies a sequence consistent with the −10 box consensus but we were not able to identify a − 35 box. To begin the characterization of the putative pyruvate-induced promoter, we built an *in-cis* β-glucuronidase (*uidA*) transcriptional fusion of the 220 bp intergenic region between the *mctP* operon and the next upstream gene and measured its activity in minimal medium supplemented with pyruvate or succinate in a parent or a Δ*rpuR* background. Congruently with the RNAseq results, the transcriptional fusion was activated in the parental background only under pyruvate-utilizing conditions but not on the Δ*rpuR* strain. When the mutant strain was complemented with *rpuR* cloned in an IPTG-inducible plasmid, activation of the promoter was restored upon induction of *rpuR* expression with IPTG (Figure 4, blue bars). To determine the minimal region necessary for RpuR-dependent regulation, we built *in-trans* transcriptional fusions from several derivatives of the *mctP* promoter and measured their activity in minimal medium supplemented with pyruvate in a parent or a ΔrpuR background. While none of the derivatives show induction in the ΔrpuR strain (data not shown) our results in the parent strain outlined a minimal inducible promoter encompassing the region between the −82 and + 1 nucleotides from the TIS (Figure 5A). Moreover, we observed a ~ 65% drop in the induction of the Pr*mctP*-75 derivative compared to the Pr*mctp*-82 derivative, but not a complete loss of induction as with other fragments. These results indicated that an important element for RpuS/R-mediated induction could be located around nucleotide −75 (Figure 5A). A closer examination unveiled an imperfect direct repeat located between the −80 and − 43 bases from the TIS (Figure 5B). To determine its relevance to the RpuS/R-mediated *mctP* induction, we performed a search for *rpuS* in the Rhizobiaceae. Our results led to the identification of four *Bradirhizobium* and five *Sinorhizobium* strains in which this gene is also neighboring a *mctP* homolog (Supplementary Figure S4). Notably, the direct repeat is conserved in the *mctP* promoter region of these strains (Figure 5B), suggesting it may play a relevant role in the regulation of the *mctP* operon. This hypothesis is strengthened when comparing the complete loss of activity of the fusion Pr*mctP*-40 in which both repeats are absent, with the reduced induction of the Pr*mctP*-72 fusion in which the cloning of the *mctP* promoter derivative for the construction of the transcriptional fusion resulted in a change of 6 bp of the first repeat, but not in its complete deletion (Figure 5B).

To further demonstrate the relevance of the direct repeat in the activation of the *mctP* promoter by the RpuS/R we built an *in-cis uidA* transcriptional fusion with a fragment of the Pr*mctP* derivative in which 4 bp of the second repeat were changed from AGTAG to TTGCA (Figure 5B, Pr*mctP*-mut2). Then, we measured its activity under pyruvate-utilizing conditions in a parent or a Δ*rpuR* background. In concordance with our previous results, the transcriptional fusion was activated in the parent and in the complemented Δ*rpuR* strain (upon induction with IPTG), but only at ~40% rate when compared with the parental promoter; as expected, no activation was detected in the Δ*rpuR* background (Figure 4, orange bars).

Finally, we used MEME to search for this motif in a 500 bp putative promoter region immediately upstream of the other top differentially expressed genes identified through our RNAseq assay (Table 2). Neither *pycar* nor the other genes contained the imperfect direct repeat in the searched region.

Altogether, our results confirm the role of the direct repeat in the pyruvate-dependent induction of the *mctP* promoter and demonstrate that this induction is contingent on the presence of the RpuS/R system.

### Deletion of CrbS or RpuS in *Sinorhizobium fredii* Affects Nodulation

**Figure 6:**
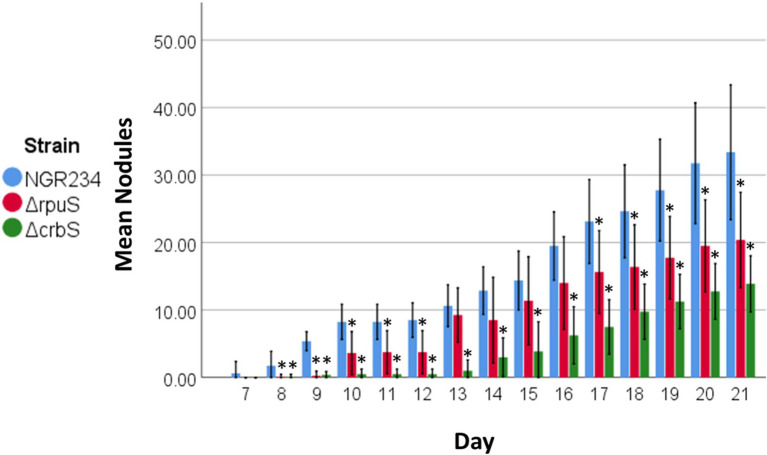
Common bean nodulation kinetics of *S. fredii* NGR234 (blue) and its Δ*rpuS* (red) and Δ*crbS* (green) derivative mutant strains. Bars represent the average number of nodules per condition per day of 8 plants ± SD per condition. An asterisk over bars indicates significant differences (*p* < 0.05).

Finally, we performed nodulation kinetic assays in beans to assess the effect that the deletion of the CrbS/R or the RpuS/R systems may have on this relevant biological process. Our results showed that the *ΔcrbS* mutant strain had a significant delay of approximately 7 days on nodulation in all the treated plants and at the end of the experiment produced ~50% fewer nodules than the parent strain. On the other hand, the deletion of *rpuS* had a milder effect on nodulation delay. Plants inoculated with the *ΔrpuS* strain showed ~33% less nodules than the parent strain after 30 days (Figure 6).

## Discussion

The study of TCSTS with SLC5/STAC histidine kinases as the sensing and signal transmission component has focused mainly on the Pseudomonadales and other Gammaproteobacteria. In this work, we have expanded the characterization of this family into the Alphaproteobacteria by identifying a homolog of the CrbS/R system and characterizing the novel RpuS/R system as a *mctP* regulator in *S. fredii* NGR234.

The identification of the RpuS/R system shows that there are two lineages of rhizobia dependent on MctP for optimal pyruvate utilization, but that these are under the control of different regulatory systems, specifically the MctS/R system and the RpuS/R system identified here. A search of the GeConT database for MctP homologs in nitrogen-fixing Alphaproteobacteria returned 26 hits (Table 3; Supplementary Figure S4). Seventeen are adjacent to a MctS/R system and are found in bacteria of the genus *Rhizobium* (family *Rhizobiaceae*). Nine systems are adjacent to a RpuS/R system (Supplementary Figure S4) and are found in bacteria belonging to the promiscuous genus *Sinorhizobium* (family *Rhizobiaceae*) and the slow-growing genus *Bradyrhizobium* (family *Nitrobacteraceae*). From the MctS/R associated hits, very few are located on the chromosome, only those from *R. tropici* and *Agrobacterium*, while from the MctS/R associated hits, all those that come from *Bradyrhizobium* are located on the chromosome; Bradyrhizobia are known for having characteristic large genomes, and very few plasmids (Ormeño-Orrillo and Martínez-Romero, 2019). From the *Sinorhizobium* genus, only strain USDA257 that is missing one repABC plasmid (Schuldes et al., 2012) has a chromosomally encoded *mctP* gene. The rest of the hits, in *Rhizobium* and *Sinorhizobium*, are located on repABC megaplasmids. In the *Rhizobiaceae*, all the plasmids with a MctS/R associated *mctP* gene belong to the p42c family, as defined by their RepC protein (González et al., 2019). Although no detailed genealogy has been determined for the plasmids of *Sinorhizobium*, it has been shown that the non-symbiotic *repABC* plasmids from the genus differ significantly from those of the *Rhizobiaceae* (Wang et al., 2018; González et al., 2019). Moreover, plasmid pNGR234b of *S. fredii* is considered a hot spot for lateral transfer because of the presence of 142 transposases, as well as the determination that ≥40% of its encoded genes are not strain-specific and were probably acquired from a wide variety of other microorganisms (Streit et al., 2004; Schmeisser et al., 2009). Overall, these observations suggest a crucial role of recombination and lateral transfer in the acquisition of the *mctP* gene by rhizobia and its association with different regulatory systems.

**Table 3:** Rhizobia with *mctP* neighbored by a TCSTS.

Strain	Replicon	Regulation
*Agrobacterium rhizogenes* K599	Chromosome	MctS/MctR
*Rhizobium tropici* CIAT 899	Chromosome	MctS/MctR
*Rhizobium sp.* IE4771	N.D.	MctS/MctR
*Rhizobium sp*. Kim5	pRetKim5d	MctS/MctR
*Rhizobium etli* N561	pRspN561a	MctS/MctR
*Rhizobium* sp. N1341	pRspN1341b	MctS/MctR
*Rhizobium leguminosarum* bv *Viciae* 3,841	pRL10	MctS/MctR
*Rhizobium leguminosarum* bv *trifolii* WSM2304	pRLG201	MctS/MctR
*Rhizobium phaseoli* N161	pRphaN161a	MctS/MctR
*Agrobacterium radiobacter* K84	Chromosome	MctS/MctR
*Rhizobium favelukesii* LPU83	pLPU83c	MctS/MctR
*Rhizobium etli* IE4803	pRetIE4803d	MctS/MctR
*Rhizobium etli* bv. *mimosae* Mim1	pRetMIM1b	MctS/MctR
*Rhizobium etli* CFN42	pCFN42c	MctS/MctR
*Rhizobium* sp. N731	pRspN731b	MctS/MctR
*Rhizobium leguminosarum* bv *trifolii* WSM1325	pR132505	MctS/MctR
*Rhizobium leguminosarum* bv. *trifolii* CB782	U.P.	MctS/MctR
*Bradyrhizobium diazoefficiens* USDA 110	Chromosome	RpuS/RpuR
*Bradyrhizobium japonicum* E109	Chromosome	RpuS/RpuR
*Bradyrhizobium japonicum* USDA6	Chromosome	RpuS/RpuR
*Bradyrhizobium ottawaense* OO99	Chromosome	RpuS/RpuR
*Sinorhizobium fredii* NGR234	pNGR234b	RpuS/RpuR
*Sinorhizobium americanum* ECFNEI73	pCFNEI73c	RpuS/RpuR
*Sinorhizobium fredii* USDA257	Chromosome	RpuS/RpuR
*Sinorhizobium fredii* HH103	pSfHH103e	RpuS/RpuR
*Sinorhizobium* sp. CCBAU05631	pSS05631b	RpuS/RpuR

N.D., Not Determined; U.P., Unnamed RepABC plasmid.

Although surprising, the finding that *mctP* is one of the genes under the control of the RpuS/R system is biologically sound. In rhizobia, the regulation of pyruvate utilization through the enzymes pyruvate carboxylase (EC 6.4.1.1) and pyruvate dehydrogenase (EC 1.2.4.1) is complex, including transcriptional and post-translational mechanisms, depending on the availability of different compounds, and varies among different organisms (Dunn, 1998; Bolger et al., 2014; Geddes and Oresnik, 2014). Therefore, acquiring a modular improvement that enhances substrate acquisition and provides an ecological advantage over other microorganisms is a straightforward addition to an intricate network that otherwise may have little flexibility for modification. These observations also highlight the adaptation of TCST sensors, particularly those belonging to the SL5/STAC family, as regulators of genes requiring early detection and fast response to subtle concentration changes of substrates in a highly competitive environment. Interestingly, the *ΔmctP, ΔrpuS*, and *ΔrpuR* strains showed remanent growth on pyruvate as the sole carbon source after 24 and 72 h of incubation in liquid and solid MM, respectively (Figure 2; Supplementary Figure S3). This observation is consistent with previous reports in *Rhizobium leguminosarum that show that ΔmctP, ΔmctS, and ΔmctR* strains were still able to grow on alanine and pyruvate, although at a lower rate (Hosie et al., 2002). These results could be explained by a leaky expression of the *mctP* promoter in the *ΔrpuS*, and *ΔrpuR* strains or by the slow assimilation of pyruvate by less efficient transporters. It is not uncommon for bacteria to have several transporters for pyruvate transport; for example, in *E. coli* at least two uptake systems and one excretion system for pyruvate have been identified (Kreth et al., 2013). Finally, the fact that we were unable to locate the imperfect direct repeat in the putative promoter region of any of the other genes that showed differential expression in our RNAseq assays suggests that they are not part of the RpuS/R regulon. Nonetheless, more experimental evidence is required to confirm this hypothesis.

Of particular interest was our finding that both the *ΔcrbS* and *ΔrpuS* strains start nodulation later than the parental strain. Several works have shown different impacts on bacteria–host interactions caused by the deletion of the SLC5/STAC TCSTS. For example, it was found that *V. cholerae* strains lacking CrbS or CrbR are less virulent when compared with the parent strain. At the same time, the equivalent mutants in *P. aeruginosa* and *P. entomophila* are fully pathogenic in *Drosophila* (Jacob et al., 2017). It was also recently reported that the root colonization efficiency of a plant growth-promoting strain of *P. aeruginosa* was severely impeded in its Δ*cbrA* and Δ*cbrB* derivatives compared to the parental strain (Sivakumar et al., 2021). Since the RpuS/R system of *R. fredii* was characterized for the first time in this work, no previous information of its involvement in nodulation is available. However, it was shown previously that in *R. leguminosarum*, a Δ*mctP* deletion mutant was still capable of forming nitrogen-fixing nodules on peas, although the authors mention no details on nodulation kinetics (Hosie et al., 2002). Overall, these observations suggest that the effects elicited by the deletion of SLC5/STAC TCSTS on host–microbe interactions depend significantly on each microorganism’s particular environmental conditions and adaptations. For *S. fredii* NGR234, a plausible explanation could be the dependence on plant exudates as a carbon source by the bacteria, of which pyruvate and acetate are relevant components (Koo et al., 2005). However, since *S. fredii* NGR234 has an extraordinary nodulation-host diversity, nodulating more than 112 genera of legumes (Pueppke and Broughton, 1999), it could be interesting to further characterize the role of the CrbS/R and RpuS/R systems in nodulation in other plant species.

Finally, the identification of the CrbS/R system in *S. fredii* and the confirmation of its participation in the regulation of acetate utilization highlight its importance in bacteria. Previously, when characterizing this system in *P. fluorescens* we failed to obtain a phenotype for the mutants on several of the genes it regulated, probably because our experimental conditions failed to reproduce the natural conditions during which their activity is important (Sepulveda and Lupas, 2017). Currently, we are characterizing the regulon of different homologs of the CrbS/R system, which also seem to be involved in the regulation of acetate utilization, in different families of bacteria with various lifestyles. We expect that this multi-organism comparative characterization will allow us to better understand not only the sensing and signaling mechanisms of the SLC5/STAC sensors, but also the metabolic role of the genes regulated by these systems.

## Data Availability Statement

The datasets presented in this study can be found in online repositories. The names of the repository/repositories and accession number(s) can be found below:https://www.ncbi.nlm.nih.gov/, SRR16684185https://www.ncbi.nlm.nih.gov/, SRR16684186https://www.ncbi.nlm.nih.gov/, SRR16684183https://www.ncbi.nlm.nih.gov/, SRR16684184.

## Author Contributions

AR, MA, GG, RH, MG, and ES constructed the plasmids and strains, performed the biochemical and microbiological assays, and analyzed the data. CC and SB performed the nodulation assays and analyzed the data. AS-F and CG-S performed the RNAseq assays. RC-R and AP analyzed the RNAseq data. AL and RC performed the bioinformatic analyses. ES, AR, RC, and AL contributed to conception and design of the study. ES, AL, and AP provided funding. ES wrote the first draft of the manuscript. RC-R, CG-S, AL, and AP wrote sections of the manuscript. All authors contributed to manuscript revision, read, and approved the submitted version.

## Funding

This work was funded by the Max Planck Society through the Max Planck Partner Group in Signal Transduction and Agricultural Microbiology, by the Mexican Research Council (SEP-CONACYT project no. A1-S-7797), and by the Knut and Alice Wallenberg Foundation grant KAW 2015.0225. RNAseq analysis was performed on resources provided by SNIC through the Uppsala Multidisciplinary Center for Advanced Computational Science (UPPMAX, Uppsala University, Sweden), *via* projects SNIC 2017-7-258, uppstore2017093, and SNIC 2020/16-261 to Andrea Puhar.

## Conflict of Interest

The authors declare that the research was conducted in the absence of any commercial or financial relationships that could be construed as a potential conflict of interest.

## Publisher’s Note

All claims expressed in this article are solely those of the authors and do not necessarily represent those of their affiliated organizations, or those of the publisher, the editors and the reviewers. Any product that may be evaluated in this article, or claim that may be made by its manufacturer, is not guaranteed or endorsed by the publisher.

## Supplementary Material

The Supplementary Material for this article can be found online at: https://www.frontiersin.org/articles/10.3389/fmicb. 2022.871077/full#supplementary-material
