# Supplementary material for: RpuS/R Is a Novel Two-Component Signal Transduction System That Regulates the Expression of the Pyruvate Symporter MctP in Sinorhizobium fredii NGR234
Source: Front Microbiol. 2022 Apr 29;13:871077. doi: 10.3389/fmicb.2022.871077 (PMC9100948; doi:10.3389/fmicb.2022.871077)
Supplement: Supplementary file 2 [file Data_Sheet_1.PDF]

```
Sfr_RpuS   -MLSGSVILASAFAYLLLLFAVASYGDRRASRSKIA---SKGRPLVYALSLAIYCTSWTYFGGVGLAAEHGLEFTGIYIGPILMFTLGMPLIRRIVRLAKTEKLTSVADFVAARYGKN
Sfr_CrbS   --MPGWVVLSVSTAYVVILFLIAWWTDRHGTGRRLAVPSEWLAALGYALTLAIYNTSWSFYGSVGRALS-GFDFLSIYVGPTLVLVFGQRLIARIVTVAKSQNITSIADFIAARYGKS
Pfl_CrbS   MTLSSGLIAAVALAYMAIMFAIAFYGDRRRAP----LP-PRVRAWVYSLSLAVYCTSWTFFGAVGQAAEQLWAFLPIYLGPVLLLVLAPWVLQKMILISKQENITSIADFIAARYGKS

Sfr_RpuS   PAVAAIVALISLVGAIPYIALQLKAVSSSVAAMIDTSDYGIGAGENFIDLPLLVTLFLACFAIVFGTRHTDATEHQDGLILAIAMESVVKLVAMLTAGIYIVFVLFDGPANLLAAAQQ
Sfr_CrbS   QALAALVTMAALLAVLPYIALQLKAVGTSFDILTSPVAAPGGTRPIRGDSTLAVAASMALFAIMFGVRHIHASEHHRGLMAAIAFESLVKLSAFVIVALFIVFGMFDGLGDLIARTQA
Pfl_CrbS   QSLAVVVALICLVGVLPYIALQLKGIVLGVNLLIGAGADTTGTRA--QDTALIVSLVLALFTIVFGTRNLDATEHHRGMVLAIAFESLVKLFAFLAVGAFVTYGLYDGFGDLFSQAIL

Sfr_RpuS   SPAVLSALEYQTPVARWILLIVLSSFGIIMLPRQFHVTVVENRTENELRTAGILFPLYLIAINLFVLPIAIAGILTFSGSGDADLYLLALPLAGDVPFITLLTFIGGFSAATAMVIVA
Sfr_CrbS   DPRMESLLAPDFADPTWISNTIIAAISFLCLPHMFHVAVVENQSLAQARAAAWLYPAYLIVFSVLMVPIAMAGVARFADTVNPDTFVISLPLAADAPLMALVAFLGGFSAATGMVIVA
Pfl_CrbS   APRLEEYWKETVNWPSMVVQTGVAMMAIICLPRQFHVTVVENIDPQDLRLAKWVFPAYLILAALFVVPIALGGKMMLPGSVLPDSYVISLPMAEAHPALAVLAFIGGASAATGMVIVA

Sfr_RpuS   SVALSIMISNDIVMPVFLRQRLGTRGTLQESMAGTLLNIRRTAIFAVLLLGYSYYRSADMSAGLASLGLLSFVAISQMAPALLGGLVWRQANARGAIAGMVSGFLVWAYALFLPSLGG
Sfr_CrbS   SVALSTMLCNDVIVPLLLRSRLVGRSGAIWPSPSALLLVRRVSVAGILLLAYMMNRLVDQAYPLTVIGLLSFVAIAQFGPAFLGGLLWRRAKGAGAVAGISIGFAFWIYTLLLPSIAP
Pfl_CrbS   SIALSTMVSNDMLLPWLLRRSSAERPFEVF--RHWMLSVRRVSIVIILLLAYVSYRLLGSTASLATIGQIAFAAVTQLAPAMLGALYWKQANRRGVFAGLAAGTFLWFYTLVLPVTAK

Sfr_RpuS   PDNSHVAS---TI--LSFLLPFTDLFSGPQSDPLVNASALSLLVNVFAYVLGSLTRTPKPLERFQAGVFITRRSRTERAFRGRKTKVTVRDLKTTIARYMGDERMQRSFHTYEQQSGR
Sfr_CrbS   LVPAVDALVANGPFGLAW-LRPQSLFGVAGLDPISHATLWSLGANLLVFAAVSLLADRSPLERLQAEAFAEGTPAP-PPLASLPLVTRLDDLKTFAARFVGAERSASAFEDFVEQRRA
Pfl_CrbS   SLGWSLS----LFPGLTW-MHSHPLG--LSVTSLTLGTVFSLAGNFTLFVWVSMLSRTRVSEHWQAGRFIGQEISQ-RASARSMLSVQISDLLSLAARFVGEERAQQSFIRFAYRQGK

Sfr_RpuS   WLDENASADMALVHFSEQLLGSAIGSSSARLVFSLVLQRMDDPSSDTAWLLDQASEALQHNQDMLQTALSQMDQGIAVFDNASNLIIWNRRFRELLDLPEAAGQVGFPLADIVAILAR
Sfr_CrbS   --RNDGLADTEAMRFTENLIAGALGAASARVVMAAALESRSLSRKAAVGMLDEASQALHFNRKLLQGALESVPQGICVFDANLAIEAWNARFLTLLDLPRDLIRVGLPLAELVDFNRE
Pfl_CrbS   GFNPNQNADNDWIAHTERLLAGVLGASSTRAVVKAAIEGREMQLEDVVRIADEASEVLQFNRALLQGAIENITQGISVVDQSLKLVAWNRRYLELFNYPDGLISVGRPIADIIRYNAE

Sfr_RpuS   RGDIRKDEEKTLVANFLALD---KPFLLE-LGNGTRIVEVRTNAMPDKGIVTTYTDITPRVAADMALKQANETLELRVAERTSELTRVNRELGEARAVAEDANIGKTRFFAAAGHDIL
Sfr_CrbS   RGEYDAEDLKALLVNR-DLATQSWPYVYERKRPDGMVLEIAYDRMAAGGYISTYTDVTERHRAAAGLRRANEELERRVQERTQALEQ-------AKAEAERANIGKTRFLAAASHDLL
Pfl_CrbS   RGLCGPGEAEVHVARRLHWMRQGRAHTSERLFPNGRVIELIGNPMPGGGFVMSFTDITAFREAEQALTEANEGLEQRVTERTHELSQLNVALTDAKGVAESASQSKTRFLAAVSHDLM

Sfr_RpuS   PLNAARLYSSSLVQERLG----------DSDNKALVQNIDSSLESVEAILGAVLDISRLDTGAMKPRLQAVPLNDLLRRIETDFAPMARAKDIAFTVMPTSLVVRSDPNLLRRVVQNL
Sfr_CrbS   QPLNAARLYLAALDESLRLKPDSAEKDLRTEERALAKNAAAALSSTERLLDELLDISSYDSGAVRAQPVDFSVGSLLAQLELEFSALARQRGLTLKVVASTLSVRTDPQLLRRVLQNL
Pfl_CrbS   QPLNAARLFSAALSHQ--------NDGLSSEARQLVQHLDSSLRSAEDLISDLLDISRLENGKINPQRQPFVLNELFDTLGAEFKALAQEQGLRFRLRGSRLRVDSDIKLLRRILQNF

Sfr_RpuS   VSNAIKYTLEGKVLVGVRQHGQTATIEVLDSGIGIPASKFRTVFKEFARLDEGAR-TASGLGLGLSIVDRISRVLNHPVGLQSKPGKGTGFKVSVPLDTSATEPARPQPVAATKTSEA
Sfr_CrbS   LSNAIRYTPRGRVLLGCRRRGDSLRIEVRDTGIGIAPERQQEIFEEFRRLDTGEE-REKGSGLGLAIVERICRLLDLPLEVRSRPGQGTCFVVTVPRGTAGAAATVSEKS-ATPSVAS
Pfl_CrbS   LTNAFRYA-DGPVLLGVRRRKGELCLEVWDRGPGIPQDKQKVIFEEFKRLDSHQTRAEKGLGLGLAIADGLCRVLD HRLSVRSWPGKGSVFSVRVPLARNQATPLVKTPQ-ETGLP-

Sfr_RpuS   LTGLAVVCIDNEPKILEGMALLLGGWGCAVTTVESVAACARMGPGNLPARPDAIVADYHLDD-GTGIEAIAAIRALWQESIPALMVTADRTPEVRGAAERDGVSLQHKPVRPAALRAW
Sfr_CrbS   HTALTVLCVDNDEAIRAGLSALLSRWGHRPIVASDDVTAL-ANC--SGQVPDLALVDYHLD-GTTGVEVLGRLRKHWGREVRGLVITADRSEAVGAKAKALACEIMAKPVKPAALRRY
Pfl_CrbS   LSGAQVLCVDNEESILIGMRSLLTRWGCEVWTATDQAQCAALLA--EGVRPQLALVDYHLDHGETGTELMGWLRAQLAEPIPGVVISADGRPEMVAEVHAAGLDYLAKPVKPAALRAL

Sfr_RpuS   LTQLAAAGRTAAE---------------
Sfr_CrbS   LNAVGLQKGAGLQRDGEEVHEHAHRHRG
Pfl CrbS   LSRHLPL---------------------
```

**Figure S1.** Alignment of NGR_RS10960 (RpuS) and NGR_RS12300 (CrbS) from *S. fredii* NGR234 with CrbS from *P. fluorescens* SBW25. Relevant features are marked as follows: Red, Transmembrane regions of the SLC5 domain; Light green, STAC domain; Orange, PAS domain; Brown, DHp domain; Dark green, CA domain; Light Blue, REC Domain. Underlined bases indicate coiled coils.

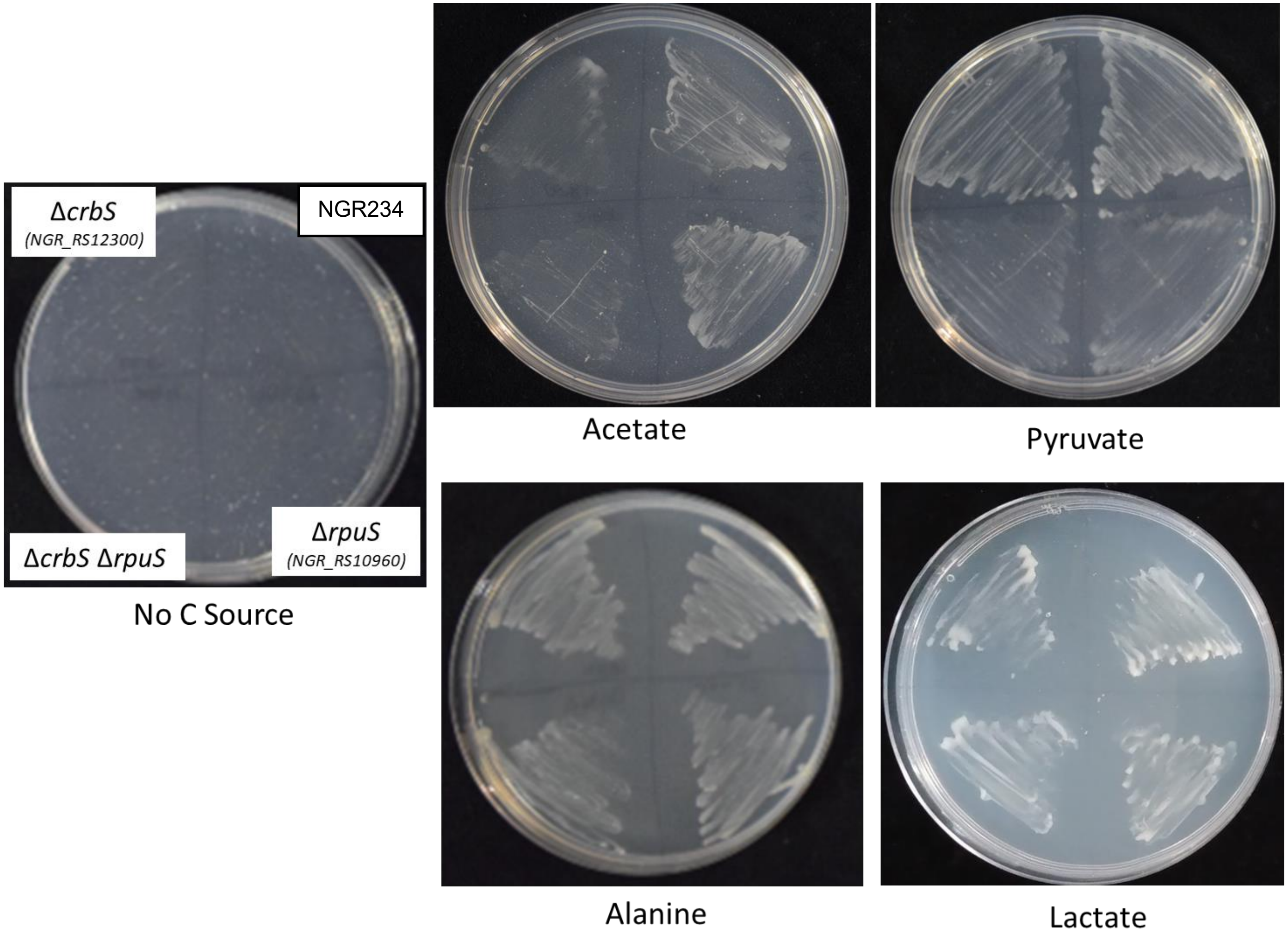


**Figure S2.** Growth of different *S. fredii* NGR234 strains in minimal media complemented with acetate, pyruvate or alanine as sole carbon source.

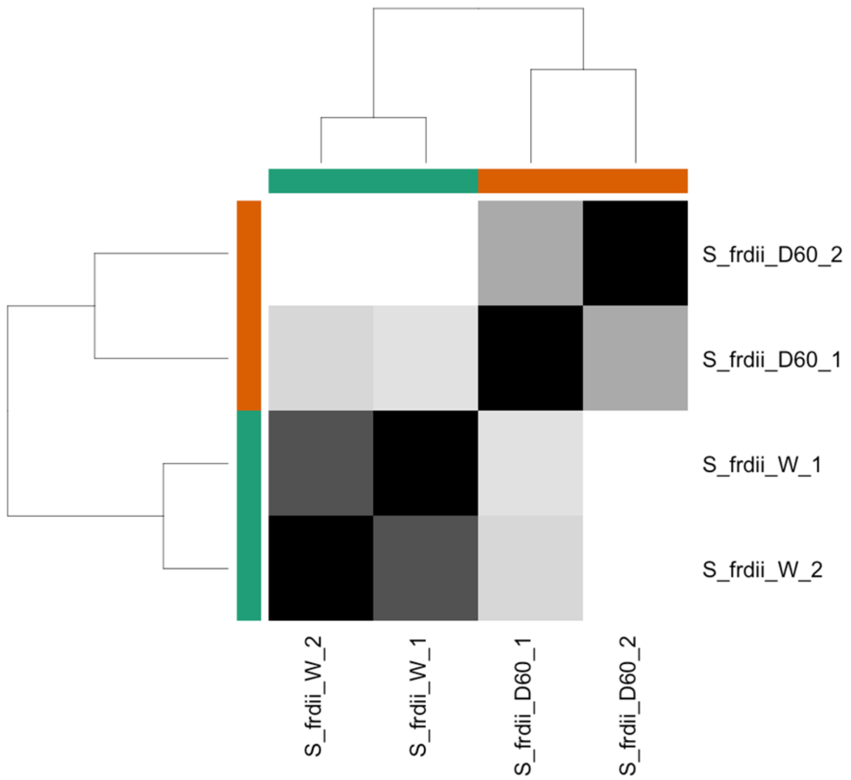


**Figure S3.** Global samples distance between RNAseq results from *S. fredii* NGR234 (S_frdii_W) and Δ*rpuS* strains (S_frdii_D60)

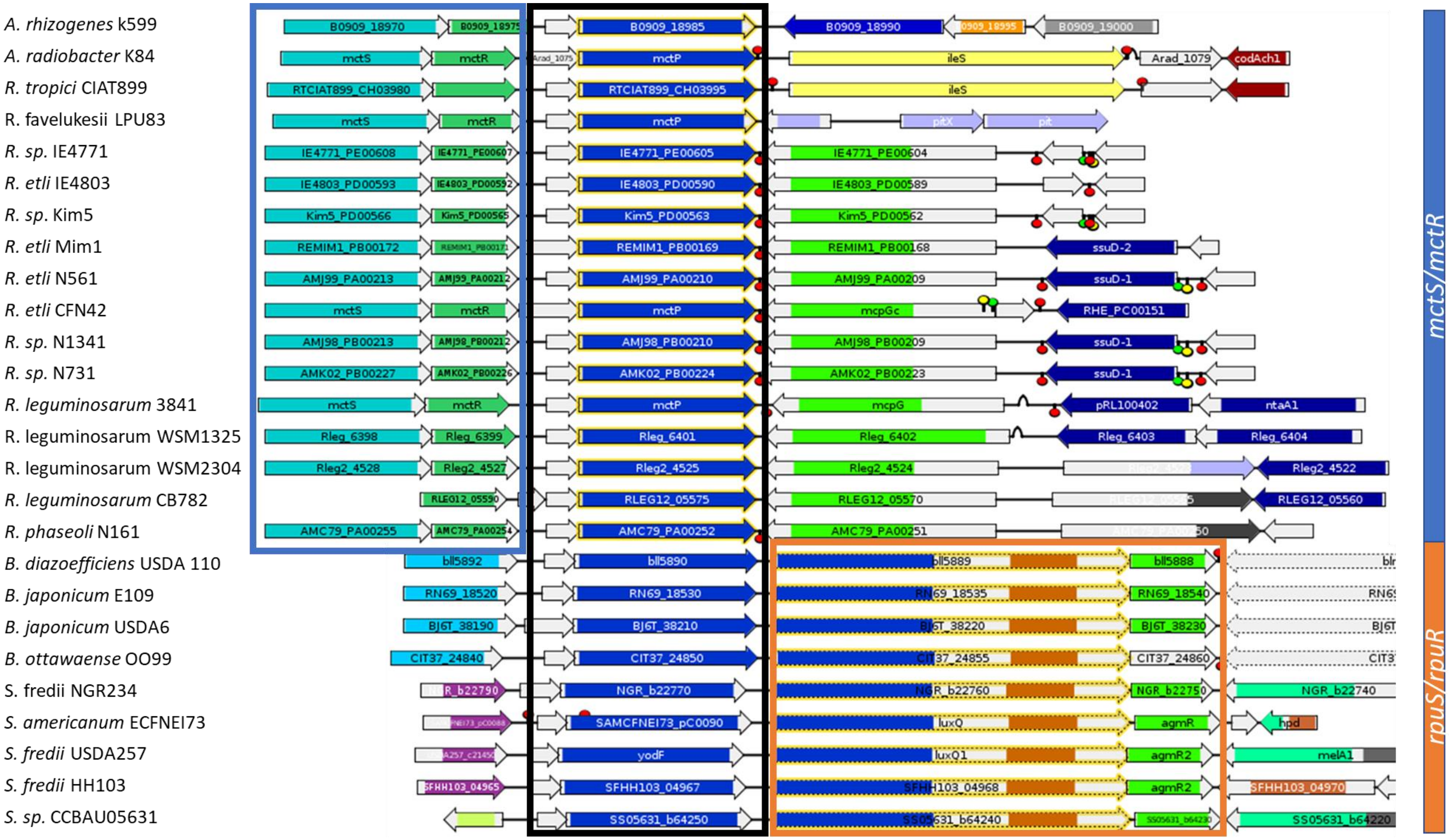


**Figure S4.** Genetic context of *mctP* (Blue arrows, black blox) in different Rhizobaceae neighbored by a *mctS/mctR* system (Blue box) or a *rpuS/rpuR* system (Orange box)

| **Primer/Usage** | **Secuence** |
|---|---|
| **Deletion of *rpuS*** | |
| 22760Au | CGACGCTGTTCAAGACGCTGTTC |
| 22760Al | GGCTGCCTGGACCATGGCACCTCGCTGCCCAACTG |
| 22760Bu | GGGCAGCGAGGTGCCATGGTCCAGGCAGCCTTGCG |
| 22760Bl | GCGCGGCTATCGCGGCTATG |
| del22760Af | TGACTTAAGCTTCCTTCTCGGCGATCGCCATC |
| del22760AR | TGACTTGAATTCGCCGCACTGAAGAAGCATCTCAG |
| **Deletion of *rpuR*** | |
| 22750u | TTGTACTAAGCTTAATCTGCTCTCGAACGCCATAC |
| 22750l | TTGTACTGAATTCTCTATTTGCTCGACGACAAGGG |
| 22750delF | CGGGTGATCATCCGCGATG |
| 22750delR | TTCTCGATGGTGCTGCACTGAA |
| **Deletion of *mctP*** | |
| mctPu | TTGTACTAAGCTTTCGATACGCGCCGTTACGC |
| mctpl | TTGTACTGAATTCATGATGGCGAAGAGCGCCATCG |
| mctPdelF | TAAGTTTGAGGGGCCGGC |
| mctPdelR | TTAGCCGTCATGACGCACGCTC |
| **Deletion of *NGR_RS10970*** | |
| Dufu | TTGTACTAAGCTTCCTCGACAAGAAGAACAAGCGGG |
| Dufl | TTGTACTGAATTCATACATGAAGGCGGCAAGCG |
| DufdelF | ATGACGGCTAACATCGACATGTCG |
| DufdelR | GTTATCCTCCCGCGATGTCTG |
| **Deletion of *crbS*** | |
| 1850Au | TTTACGCGCCTAGAGCGGGATGA |
| 1850Al | AAATTCCGCGTCCCGGCCTCACTCCTCGACGGTGAG |
| 1850Bu | GTCGAGGAGTGAGGCCGGGACGCGGAATTTGCC |
| 1850Bl | TCGTCGAGGCCGGCGATTT |
| del1850AF | TGACTTAAGCTTCCCGCATCCCGCTCTAACCTAT |
| del1850AR | TGACTTCCCGGGGATGCTTCTCGATCTCACCATGCC |
| **Deletion of *crbR*** | |
| 1840u | TTGTACTAAGCTTTTTCGATCGTCGACCGCATC |
| 1840l | TTGTACTGAATTCAATGAAGCCGTCGGCGAGAT |
| 1840del1 | GATGACGCTCCCTCCCAAGAG |
| 1840del2 | TAAGCGGTAACAACTGCGGACG |
| **Cloning of *rpuS* in pSRKgm** | |
| pSRKRpusF | CGCATCGTCATCGCGGATGAAAGCTTATCGATACCGTCGA |
| pSRKRpusR | AGGACGACCCAACCGGGCATATGCTGTTTCCTGTGTGAAA |
| RpuSpSRKF | TTTCACACAGGAAACAGCATATGCCCGGTTGGGTCGTCCT |
| RpuSpSRKR | TCGACGGTATCGATAAGCTTTCATCCGCGATGACGATGCG |

| | |
|---|---|
| **Cloning of *rpuR* in pSRKgm** | |
| 22750NdeI | TTGTACTCATATGGTCCAGGCAGCCTTG |
| 22750HindIII | TTGTACTAAGCTTCTATCCCGCATCGCGG |
| **Cloning of *crbS* in pSRKgm** | |
| pSRKCrbsF | GACGGACGGCGGCGGAGTAGAAGCTTATCGATACCGTCGA |
| pSRKCrbsR | ATGACCGAACCCGAAAGCATATGCTGTTTCCTGTGTGAAA |
| CrbSpSRKF | TTTCACACAGGAAACAGCATATGCTTTCGGGTTCGGTCATTCT |
| CrbSpSRKR | TCGACGGTATCGATAAGCTTCTACTCCGCCGCCGTCCGTC |
| **Cloning of *crbR* in pSRKgm** | |
| 1840NdeI | TTGTACTCATATGCCCGACATGACCATCAT |
| 1840HindIII | TTGTACTAAGCTTTTAAGCCGCCGCCACGCT |
| ***In-Cis* transcriptional fusions of the NGR_RS10970-mctP promoter** | |
| prVMGsalF | CTTTAGGTCGACCACGGCTCGAACGAACCGGA |
| prVMGBamR | TAGACGGGATCCGTTATCCTCCCGCGATGTCTGGA |
| ***In-trans* transcriptional fusions of the NGR_RS10970-mctP promoter** | |
| pr53gBcuF | CTTTAGACTAGTCACGGCTCGAACGAACCGGA |
| pr53gBamRb | TAGACGGGATCCGTTATCCTCCCGCGATGTCTG |
| **Mapping of the NGR_RS10970-mctP promoter** | |
| pr+18 | GATCACGGATCCGTGTGTCTCTCGAATAGCTCCAAACGC |
| pr-20 | GATCACGGATCCTAGCTCCAAACGCCGCGCC |
| pr-75 | CTTTAGACTAGTAAGGGGGGCGCCGGAGC |
| pr-82 | CTTTAGACTAGTAATGAGCGAAAGTAGGGGGGC |
| pr-30 | CTTTAGACTAGTTTGGAGCTATTCGAGAGACACACGTG |
| pr-40 | CTTTAGACTAGTGGCGCGGCGTTTGGAGCTA |
| **Mutagenesis of the NGR_RS10970-mctP promoter to PrmctP-Mut2** | |
| prMut2U | GCGCCGCGGCCTCC***TGCAA***TTCGCTCCGGCGCCCC |
| prMut2L | GGGGCGCCGGAGCGAA***TTGCA***GGAGGCCGCGGCGC |

**Table S1**. Restriction sites are underlined. ***Bold Italics*** indicate introduced nucleotide changes for mutagenesis

| | GenBank | | | | UniProt | |
|---|---|---|---|---|---|---|
| ***S. fredii* NGR234** | **Accession** | **(formerly)** | **Gene Name** | **(formerly)** | **Accession** | **Gene Name** |
| ***rpuS*** | WP_015888335.1 | YP_002824474.1 | *NGR_RS10960* | *NGR_b22760* | C3KN91_SINFN | *NGR_b22760* |
| ***rpuR*** | WP_015888334.1 | YP_002824473.1 | *NGR_RS10955* | *NGR_b22750* | C3KN90_SINFN | *NGR_b22750* |
| ***crbS*** | WP_012706770.1 | YP_002824738.1 | *NGR_RS12300* | *NGR_c01850* | C3MFT4_SINFN | *NGR_c01850* |
| ***crbR*** | WP_012706769.1 | YP_002824737.1 | *NGR_RS12295* | *NGR_c01840* | C3MFT3_SINFN | *NGR_c01840* |
| ***P. fluorescens* SBW25** | | | | | | |
| ***crbS*** | WP_017337284.1 | | *PFLU_RS21935* | *PFLU_4471* | C3K0X7_PSEFS | *PFLU_4471* |
| ***crbR*** | WP_012722526.1 | | *PFLU_RS05910* | *PFLU_1195* | C3KDG5_PSEFS | *PFLU_1195* |

**Table S2.** Equivalencies of identifiers for the RpuS and CrbS genes and proteins from *S. fredii* NGR234 and *P. fluorescens* SBW25 among different databases
